# Rab27a promotes degradation of West Nile virus E protein in the lysosome

**DOI:** 10.1016/j.isci.2024.109539

**Published:** 2024-03-19

**Authors:** Shintaro Kobayashi, Seira Kawai, Yukine Fukuda, Haruto Eguchi, Keisuke Maezono, Passawat Thammahakin, Hirofumi Sawa, Hiroaki Kariwa

**Affiliations:** 1 Laboratory of Public Health, Faculty of Veterinary medicine, Hokkaido University, N18, W9, Kita-ku, Sapporo, Hokkaido 060-0818, Japan; 2 Hokkaido University, Institute for Vaccine Research and Development (HU-IVReD), Sapporo, Hokkaido 001-0021, Japan; 3 Division of Molecular Pathobiology, International Institute for Zoonosis Control, N20, W10, Kita-ku, Sapporo, Hokkaido 001-0020, Japan; 4 One Health Research Center, Hokkaido University, Sapporo, Hokkaido 001-0020, Japan; 5 International Collaboration Unit, International Institute for Zoonosis Control, Hokkaido University, Sapporo, Hokkaido 001-0020, Japan; 6 Global Virus Network, Baltimore, MD 21201, USA

**Keywords:** Virology, Cell biology

## Abstract

Rab27a, a Rab family small GTPases, plays an important role in the trafficking and secretion of the intracellular proteins and has been reported to promote various viral multiplication. However, whether Rab27a is involved in West Nile virus (WNV) multiplication is unknown. This study examined the ability of Rab27a to suppress WNV multiplication. The inhibition of Rab27a expression increased viral multiplication and the intracellular levels of WNV structural proteins, E and prM proteins. Rab27a partially colocalized with E protein, mainly in the perinuclear region, while inhibition of Rab27a expression resulted in diffuse subcellular localization of E protein. In addition, some of the perinuclear E protein colocalized with the lysosomal marker LAMP1, and inhibition of lysosomal acidification increased intracellular levels of Rab27a and E proteins. These observations suggested that Rab27a inhibits WNV multiplication by inducing the degradation of viral protein in lysosomes.

## Highlights

• Rab27a inhibits WNV multiplication and the levels of intracellular E protein
• Inhibition of Rab27a results in diffuse subcellular localization of E protein
• Inhibition of lysosomal degradation increases E protein and Rab27a levels

## Graphical abstract

**Figure:**
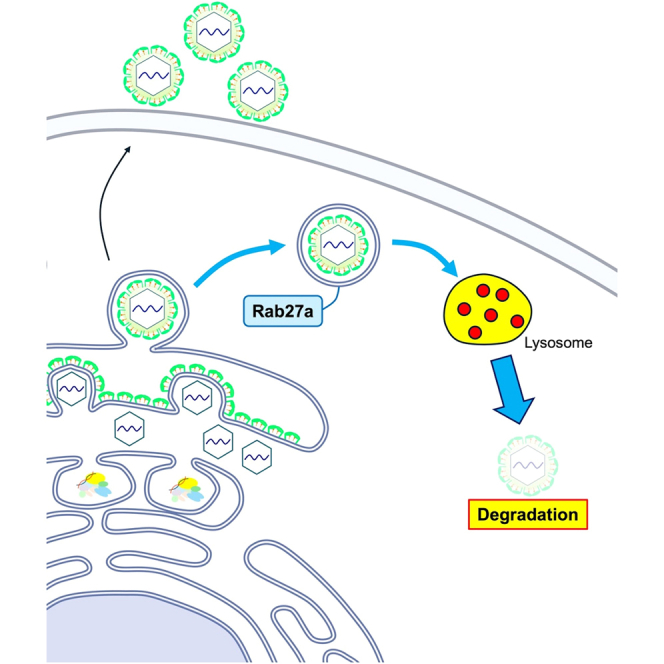

## Introduction

West Nile virus (WNV) is a positive-sense, single-stranded RNA virus that belongs to the family *Flaviviridae*, genus *Orthoflavivirus*, species *Orthoflavivirus nilense*.^1^ In nature, WNV is maintained in a cycle between mosquitoes and birds and is transmitted to other animals by mosquitoes.^2^ Humans and horses are considered to be incidental dead-end hosts and suffer serious disease, such as encephalitis and neurological complications.^2^ WNV is widespread, with evidence of its activity obtained on all continents except Antarctica.^3^ Human cases of WNV infection have been increasing in recent years, especially in Europe, and pose a risk to global health.^4^ Although vaccines are available for use in horses, no vaccine has yet been approved for use in humans, due to the requirement for multiple primary doses and an annual booster.^4^ Furthermore, no specific treatments of WNV disease are presently available. Therefore, investigations of the mechanism of viral replication and the pathogenesis of WNV infection are needed to develop vaccines and effective treatments for use in human.

WNV is internalized into mammalian host cells through receptor-mediated endocytosis.^5^^,^^6^ The decrease in pH in the endosome triggers viral and host membrane fusion to release the viral RNA.^5^^,^^6^ The viral polyprotein is synthesized using viral RNA as a template at the endoplasmic reticulum (ER) and is cleaved by host and viral proteases into the structural proteins [capsid (C), precursor membrane (prM), and envelope (E)], and nonstructural proteins (NS1, NS2A, NS2B, NS3, NS4A, NS4B, and NS5).^7^ Viral genome replication occurs in replication organelles consisting of viral nonstructural proteins, host proteins, and the ER membrane.^8^ Immature virions are assembled in the ER and transported through the host secretory pathway.^8^ Virion maturation occurs in the trans-Golgi network by furin-mediated cleavage of the prM to M, and mature virions are released by exocytosis.^8^

Rab proteins are small GTPases belonging to the Ras GTPase superfamily. They are involved in regulating membrane traffic, such as vesicle formation, motility, fusion with target membranes, and exosome release.^9^ There are more than 60 Rab proteins in humans, mutations or dysfunctions of which are related to neurological diseases, such as Charcot-Marie-Tooth Type 2B, Parkinson’s disease, and Alzheimer’s disease.^10^^,^^11^ Rab proteins are also involved in infection by various viruses. Previous studies showed that Rab5 was required for cellular entry of flaviviruses, such as Dengue virus, Japanese encephalitis virus, and WNV.^12^^,^^13^ Rab14 was shown to support Ebola virus infection by transporting VP40 protein to the plasma membrane, and to be involved in Classical Swine Fever virus infection by promoting virus assembly and maturation.^14^^,^^15^

A previous study using small interfering RNA (siRNA) screening to identify Rab proteins involved in the release of WNV particles showed that Rab8b was related to the transportation of WNV particles from recycling endosomes to the plasma membrane.^16^ Furthermore, downregulation of Rab27a promoted WNV particle release, but the detailed underlying mechanisms were unclear.^16^ Rab27a has several functions, including docking of multivesicular bodies to the plasma membrane, inhibition of phagocytosis, secretion of insulin, and trafficking and secretion of several lysosome-related organelles, including melanosomes.^17^^,^^18^^,^^19^^,^^20^ Hepatitis C virus (HCV), Hepatitis E virus, and Enterovirus A71 were reported to release their virions via a Rab27a-dependent pathway.^21^^,^^22^^,^^23^ Rab27a was shown to promote assembly of human immunodeficiency virus (HIV) and human cytomegalovirus,^24^^,^^25^ and to support HCV genome replication by interacting with HCV core protein around lipid droplets.^26^ Our previous findings on the role of Rab27a in WNV infection stand in contrast to those that implicated Rab27a in facilitating the replication of several viruses. Therefore, determination of the negative effect of Rab27a in WNV infection is needed to understand the multifaceted involvement of Rab27a in viral replication processes.

Mutations of the gene encoding Rab27a cause type 2 Griscelli syndrome in human, a rare autosomal recessive pigmentation disorder that is associated with severe immunological defects, caused by the impaired function of neutrophil and natural killer cells and the dysfunction of cytotoxic T-lymphocytes.^27^^,^^28^^,^^29^ Moreover, Rab27a was shown to play an important role in the regulation of the tumor microenvironment as an essential protein for vesicle exocytosis and exosome release, while Rab27a expression levels correlate positively with a poor prognosis in cancer patients.^30^^,^^31^ Because Rab27a is associated with viral infection, investigation of its function may provide insights into the pathogenesis of the resulting diseases and lead to novel treatment.

In this study, the roles of Rab27a in WNV infection were analyzed by using an siRNA-mediated knockdown approach. The mechanisms underlying Rab27a-mediated inhibition of WNV particle release were analyzed using Rab27a-downregulated WNV-infected cells. This study demonstrated that Rab27a is involved in the degradation of WNV E protein in lysosomes.

## Results

### Rab27a inhibits WNV multiplication

**Figure 1:**
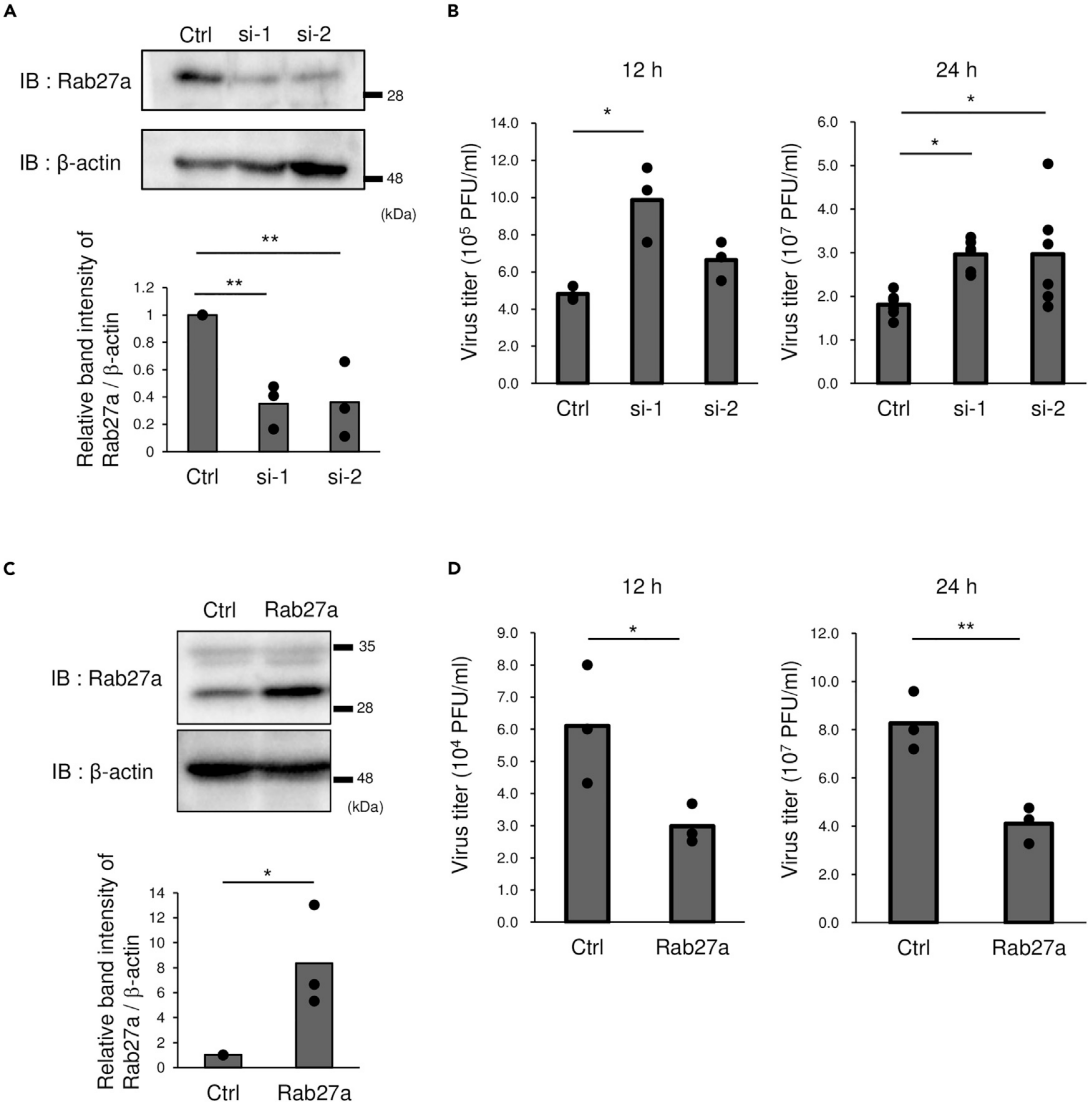
Effects of Rab27a on WNV multiplication (A) SH-SY5Y cells were transfected with siRNA for Rab27a, and Rab27a expression was assessed by immunoblotting at 1 dpt. The graph shows the relative band intensity of Rab27a/β-actin compared with the siRNA control. Bar: mean, circle: value from three independent experiments (∗∗p < 0.01, by Mann–Whitney *U* test). (B) siRNA-transfected cells were infected with WNV at 1 pfu/cell for 12 or 24 h. Viral titers were measured by plaque assay. Bar: mean, circle: value from three independent experiments (∗p < 0.05, by Tukey–Kramer test). (C) The Rab27a expression of SH-SY5Y cells stably expressing Rab27a without WNV infection was measured by immunoblotting. The graph shows the relative band intensity of Rab27a/β-actin compared with the siRNA control. bar; mean, circle; value of three independent experiments (∗p < 0.05 by Scheffe’s *F* test). (D) Cells stably expressing Rab27a or control cells were infected with WNV at 0.5 pfu/cell for 12 or 24 h. Viral titers were determined by plaque assay. Bar: mean, circle: value from three independent experiments (∗p < 0.05, ∗∗p < 0.01, by Student’s *t* test).

WNV is a neurotropic virus. Thus, to examine the effects of Rab27a on WNV multiplication, two different siRNAs for Rab27a were introduced into human neuroblastoma SH-SY5Y cells, and the growth of WNV was analyzed in these cells. The expression of Rab27a was decreased by siRNA treatment (Figure 1A). The viral titer of siRNA No.1-pretreated cells was significantly increased at both 12 and 24 h post-infection (hpi), and that of siRNA No.2-pretreated cells showed a significant increase at 24 hpi compared to controls (Figure 1B). Then, cells stably expressing Rab27a were produced and viral growth was analyzed (Figures 1C and 1D). The viral titers at both 12 and 48 hpi were significantly decreased in the cells stably expressing Rab27a compared to mock transfected controls (Figure 1D). These results indicated that Rab27a inhibited WNV multiplication.

### Rab27a decreases the levels of intracellular WNV E protein

**Figure 2:**
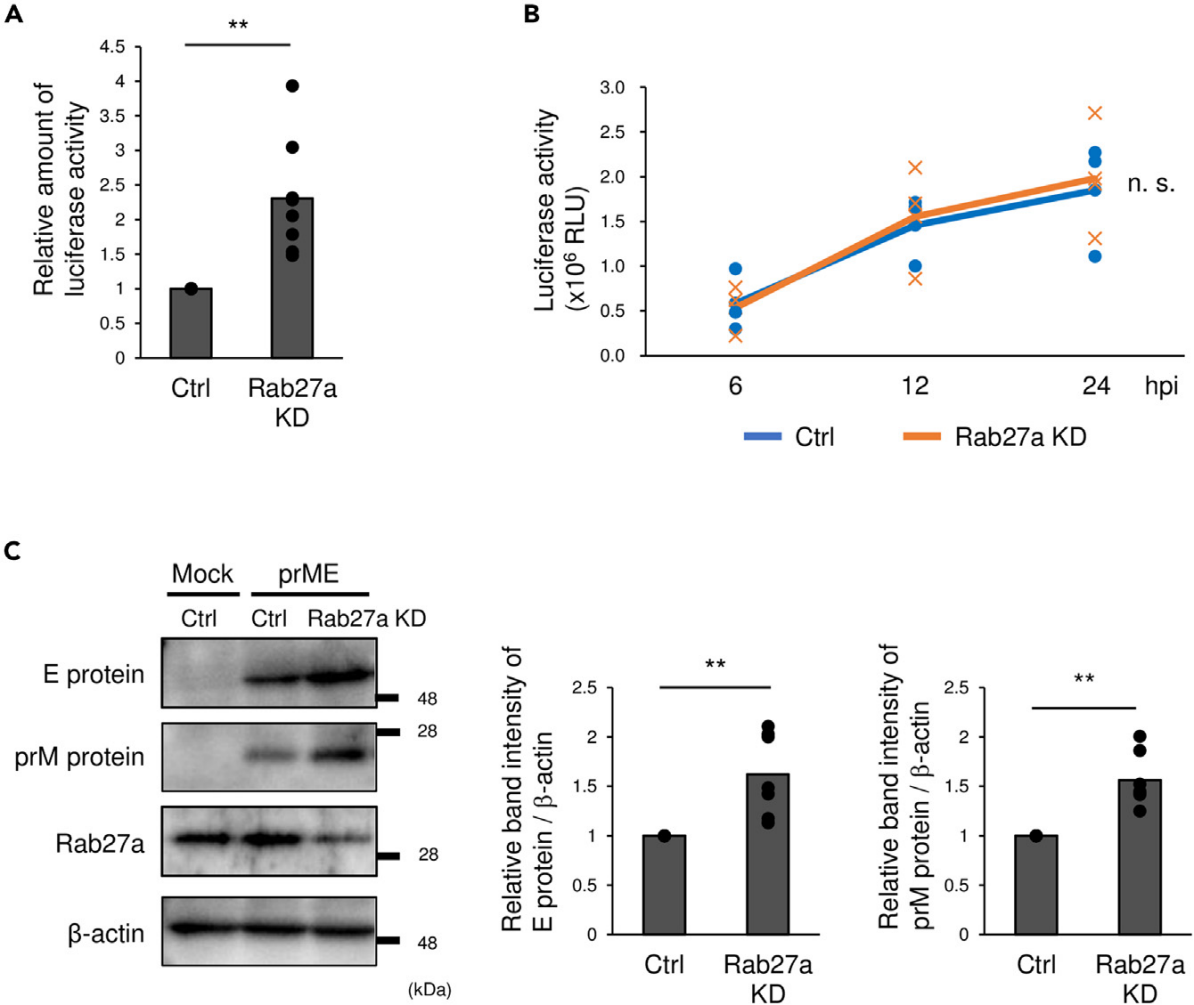
Analysis of WNV particle production in Rab27a-inhibited cells (A) SH-SY5Y cells were transfected with siRNA for Rab27a (si-1) or the control. After 1 day, these cells were transfected with plasmids expressing C protein, prME protein, and replicon. The supernatants were harvested at 48 h and inoculated into Vero cells. Luciferase activity was measured at 48 h. Bar: mean, circle: value from eight independent experiments (∗∗p < 0.01, by Mann–Whitney *U* test). (B) siRNA-introduced cells were transfected with WNV replicon RNA and the luciferase activity was measured as relative light units (RLUs) at 6, 12, and 24 h by luciferase assay. Line: mean, circle and cross; value from three independent experiments (n. s. = not significant by Student’s *t* test). (C) siRNA introduced cells were transfected with plasmid expressing prME and harvested at 48 hpt. Intracellular levels of E or prM protein were analyzed by immunoblotting. The graph showed the relative band intensity of E or prM protein/β-actin compared with siRNA control. Bar: mean, circle: value from seven independent experiments (∗∗p < 0.01, by Mann–Whitney *U* test).

Rab27a was shown previously to be related to the release of WNV particles.^16^ The effects of inhibition of Rab27a on the release of WNV particles were confirmed by examining the production of virus-like particles (VLPs). SH-SY5Y cells treated with siRNA for Rab27a were transfected with plasmids for the production of VLPs expressing luciferase in infected cells. The supernatants of transfected cells were collected at 48 h post-transfection (hpt) and inoculated into Vero cells; luciferase activity was measured at 48 h. The luciferase activity was significantly higher in the cells inoculated with the supernatant from Rab27a knockdown (KD) cells than controls (Figure 2A). Next, self-replicating WNV replicon RNA encoding luciferase was introduced into Rab27a KD cells to assess the effects of Rab27a on WNV genome replication. There were no significant differences in luciferase activity between control and Rab27a KD cells at 6, 12, or 24 hpt (Figure 2B). Finally, Rab27a KD cells were transfected with plasmid containing prME to analyze the effects of Rab27a on intracellular WNV structural protein levels. The intracellular levels of prM protein and E protein increased in Rab27a KD cells (Figure 2C). Taken together, these results suggested that Rab27a decreased the intracellular WNV structural protein levels.

### Intracellular localization of E protein is affected by Rab27a

**Figure 3:**
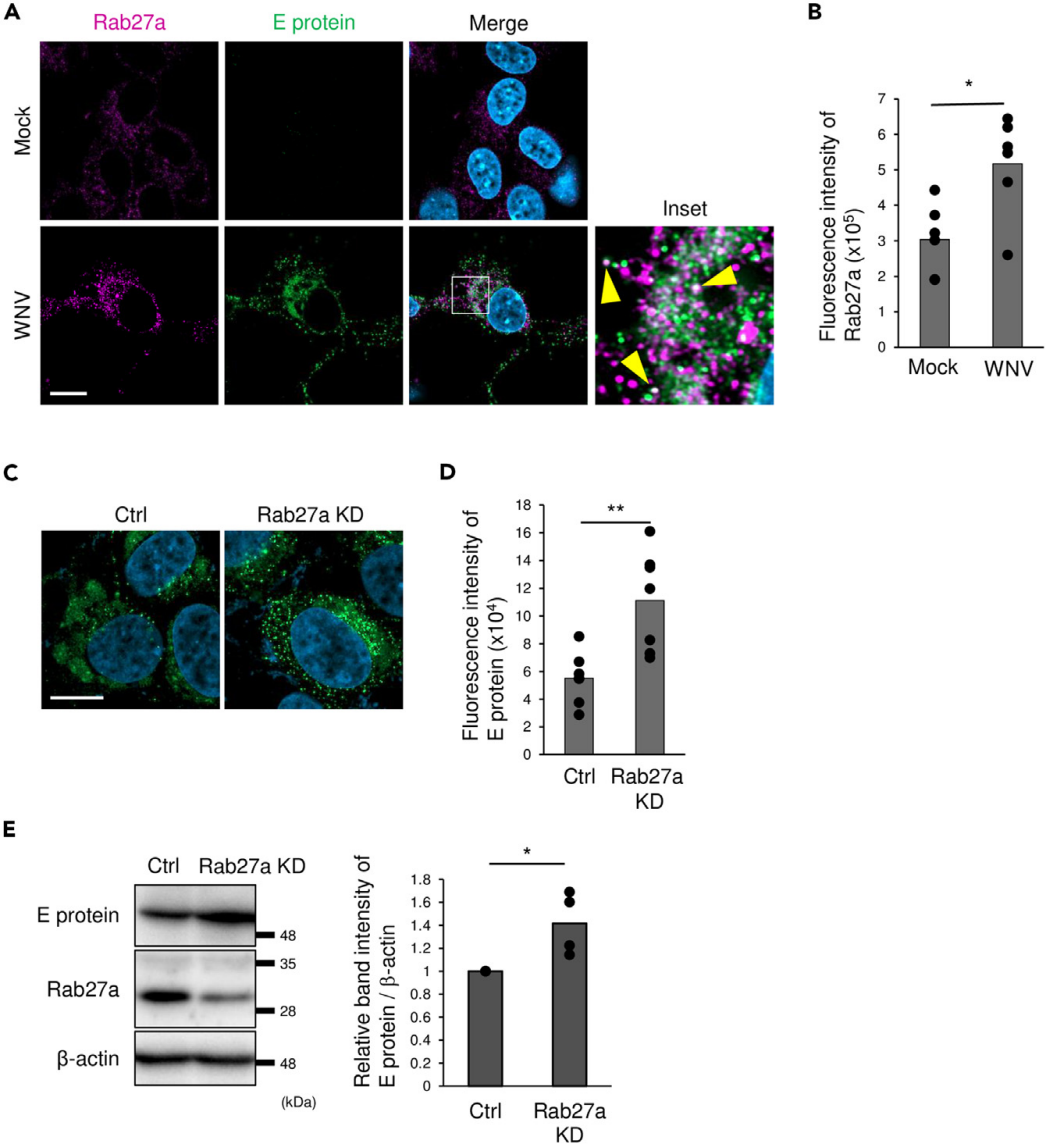
Analysis of intracellular localization of E protein and Rab27a (A) SH-SY5Y cells were infected with WNV at 1 pfu/cell. At 48 hpi, cells were fixed, permeabilized, and stained for Rab27a (magenta), WNV E protein (green), and nuclei (blue). Yellow arrowheads show colocalization of Rab27a and WNV E protein (scale bar: 10 μm). (B) The fluorescence intensity of Rab27a in each cell in mock and WNV-infected cells were analyzed using Fiji software (http://fiji.sc/Fiji). Bar: mean, circle: value from six independent experiments (∗p < 0.05, by Mann–Whitney *U* test). (C) Control cells or siRNA-transfected cells were infected with WNV at 1 pfu/cell and cultured for 48 h. The cells were fixed, permeabilized, and stained for E protein (green) and nuclei (blue) (scale bar: 10 μm). (D) The fluorescence intensity of E protein in each cell was analyzed using Fiji software (http://fiji.sc/Fiji). Bar: mean, circle: value from seven independent experiments (∗∗p < 0.01, by Mann–Whitney *U* test). (E) siRNA-introduced cells were infected with WNV at 1 pfu/cell and harvested at 48 hpi. The intracellular level of E protein was analyzed by immunoblotting. The graph showed the relative band intensity of E protein/β-actin compared to siRNA control. Bar: mean, circle: value from four independent experiments (∗p < 0.05, by Mann–Whitney *U* test).

To examine the roles of Rab27a in E protein expression, we analyzed the intracellular localization of Rab27a and E protein in WNV-infected cells. The fluorescence intensity of Rab27a in WNV-infected cells was significantly stronger than in mock-infected cells (Figures 3A and 3B), and Rab27a partially colocalized with E protein (Figure 3A). Next, the effects of Rab27a inhibition on E protein localization were examined. Diffusion of granular E protein throughout the cytoplasm was observed (Figure 3C), and the fluorescence intensity of E protein was significantly increased in Rab27a KD cells compared with controls (Figure 3D). The intracellular level of E protein was increased in Rab27a KD cells (Figure 3E). These results suggested that Rab27a was induced upon WNV infection and affected the intracellular level and localization of E protein.

### Rab27a localizes E protein to lysosomes

**Figure 4:**
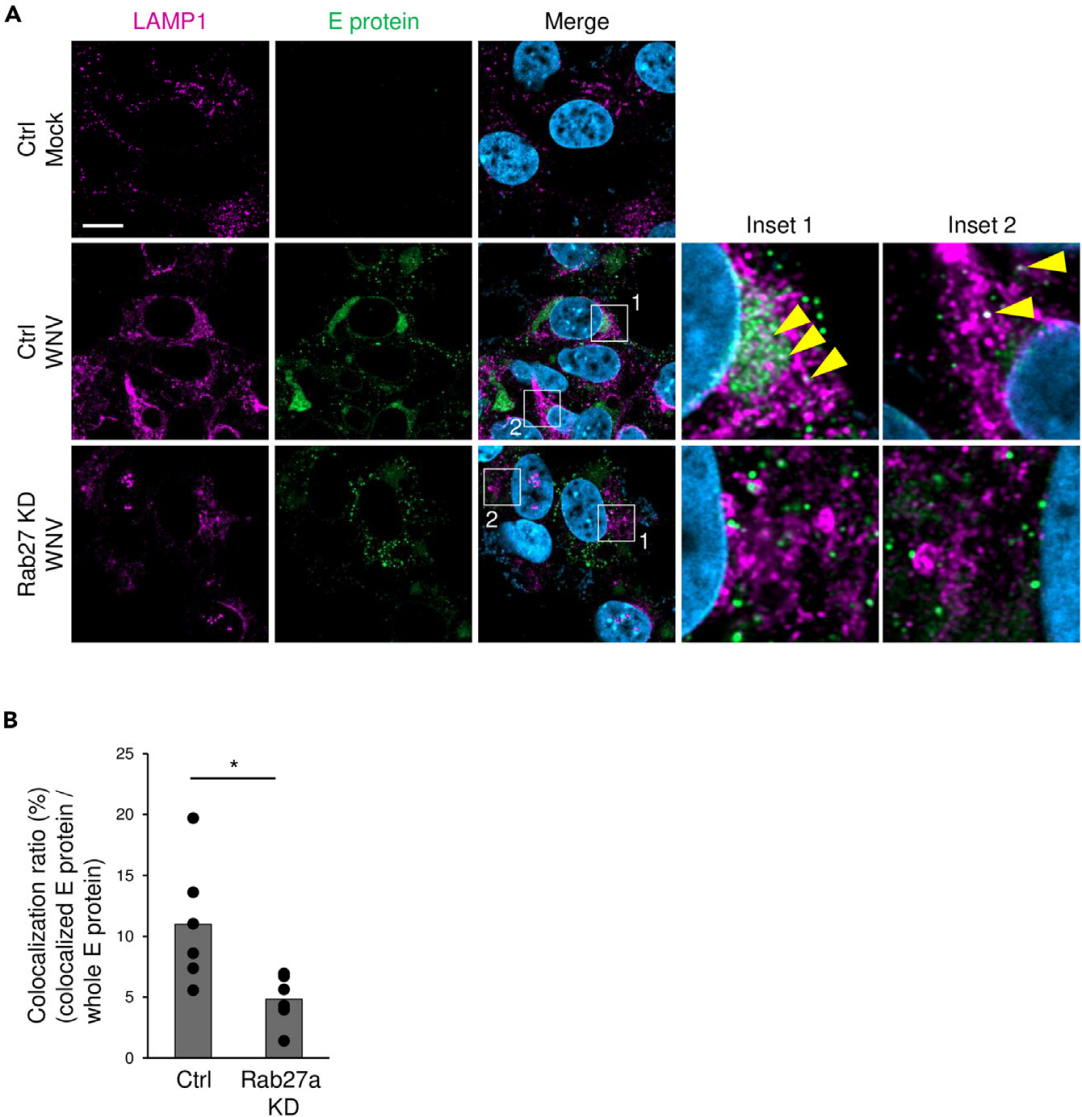
Analysis of relationships among Rab27a, E protein, and LAMP1 in WNV-infected cells (A) Control or Rab27a siRNA-transfected cells were infected with WNV at 1 pfu/cell and cultured for 48 h. The cells were fixed, permeabilized, and stained for LAMP1 (magenta), E protein (green), and nuclei (blue). Yellow arrowheads show the colocalization of E protein and LAMP1 (scale bar: 10 μm). (B) Quantification of colocalization of E protein and lysosomes. The pixel number of colocalized E protein and LAMP1 to the whole pixel number of E protein was quantified using Fiji software (http://fiji.sc/Fiji). Bar: mean, circle: value from six independent experiments (∗p < 0.05, by Mann–Whitney *U* test).

As it affected the intracellular level and localization of E protein, Rab27a was considered to be involved in the degradation of E protein. Therefore, the effects of Rab27a inhibition on the localization of E protein in the lysosome were examined. Immunofluorescence analysis showed that lysosomal-associated membrane protein (LAMP1) was partially colocalized with E protein in WNV-infected cells treated with control siRNA (Figure 4A). However, the colocalization of E protein and LAMP1 was decreased in WNV-infected cells treated with Rab27a siRNA compared to control siRNA (Figures 4A and 4B). These results suggested that Rab27a induced localization of E protein to the lysosome.

**Figure 5:**
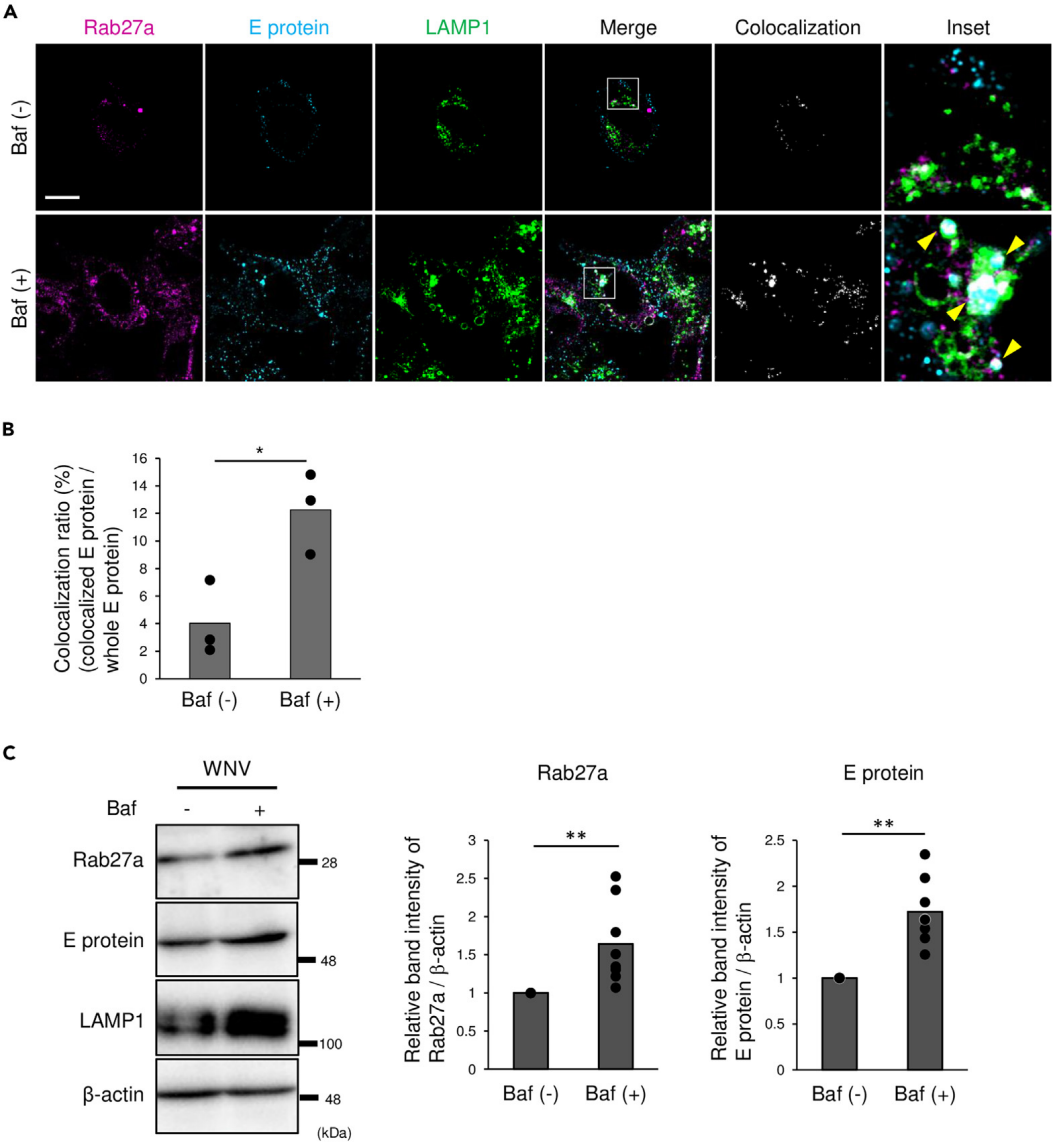
Effects of lysosome inhibition on intracellular localization and expression of Rab27a, E protein, and LAMP1 in WNV-infected cells (A) SH-SY5Y cells were infected with WNV at 1 pfu/cell. After 45 h, the cells were treated with 50 nM bafilomycin A1 (Baf) for 3 h followed by fixation and permeabilization. The results showed colocalization of Rab27a, E protein, and LAMP1. The cells were stained for Rab27a (magenta), E protein (blue), and LAMP1 (green). Yellow arrowheads indicate the colocalization of Rab27a, E protein, and LAMP1 (scale bar: 10 μm). (B) Quantification of colocalization of Rab27a, E protein, and LAMP1. The pixel number of colocalized E protein to the whole pixel number of E protein was quantified using Fiji software (http://fiji.sc/Fiji). Bar: mean, circle: value from three independent experiments (∗p < 0.05, by Student’s *t* test). (C) SH-SY5Y cells were infected with WNV at 1 pfu/cell. After 45 h, the cells were treated with 100 nM Baf for 3 h and harvested. Rab27a, E protein, and LAMP1 expression was analyzed by immunoblotting. Graphs showed relative band intensities of Rab27a or E protein/β-actin compared to Baf (−) cells. Bar: mean, circle: value from eight independent experiments (∗∗p < 0.05, by Mann–Whitney *U* test).

Next, the effects of lysosome inhibition on the localization of Rab27a and E protein were analyzed using bafilomycin A1 (Baf), which inhibits protein degradation by preventing acidification of lysosomes. Baf treatment increased the fluorescence intensity of E protein, which was colocalized with Rab27a and LAMP1 (Figure 5A). Colocalization of E protein, Rab27a, and LAMP1 was significantly increased in Baf-treated cells compared to untreated controls (Figure 5B). Next, the effects of lysosome inhibition on the levels of intracellular Rab27a and E protein were examined. The levels of intracellular Rab27a and E protein were significantly increased in WNV-infected cells treated with Baf-treatment compared to untreated controls (Figure 5C). These results suggested that E protein was degraded in lysosomes along with Rab27a.

## Discussion

A previous study showed that inhibition of Rab27a resulted in an increase in release of WNV VLPs.^16^ The present study was performed to examine the relation between Rab27a and WNV infection in greater detail. This study showed that inhibition of Rab27a promoted WNV multiplication and increased the levels of intracellular E protein, and that inhibition of lysosomal degradation resulted in increase in E protein and Rab27a levels. The main function of Rab proteins is to regulate vesicle transport in cells.^9^ Rab27a plays a role in the fusion of multivesicular bodies to the plasma membrane and in exosome secretion.^25^^,^^32^ Moreover, Rab27a is involved in the transport and secretion of lysosome-related organelles, and was shown to mediate lysosomal localization.^20^^,^^33^^,^^34^^,^^35^ These findings suggested a novel role for Rab27a in inhibition of viral multiplication by transporting WNV viral proteins to lysosomes for degradation.

Rab proteins are localized on the membrane surface of vesicles and regulate internal protein transport.^36^ Various Rab proteins, including Rab5, Rab8, and Rab11, have been shown to mediate vesicular transport of flavivirus particles.^12^^,^^13^^,^^16^^,^^37^ The simultaneous expression of flavivirus prM and E proteins in mammalian cells leads to the production of sub-viral particles, which have biological properties similar to authentic virions.^38^^,^^39^ After assembly in the ER, WNV virions, in the form of particles, are transported to the plasma membrane by vesicle transport.^8^ In this study, Rab27a inhibition was shown to result in an increase in the level and granular intracellular distribution of E protein in WNV-infected cells. In addition, Rab27a inhibition increased the expression levels of prM and E protein in the cells. These finding suggested that Rab27a transports these viral proteins into the lysosomal compartment in the form of particles. After assembly in the ER, flavivirus virions are transported to the plasma membrane via the Golgi apparatus.^8^ Identification of the original organelles of the vesicles transported by Rab27a to the lysosome provided insight into the detailed function of Rab27a in vesicular transport.

The Rab27a fluorescence signal was increased and a portion of Rab27a was colocalized with E protein in cells infected with WNV. It has been reported that upregulation of Rab27a contributes to the progression and malignancy of cancer cells.^40^^,^^41^ Increased expression of Rab27a in colon cancer cells in response to the NF-kB related inflammatory signaling pathway was shown to promote tumorigenesis.^42^ As this signaling pathway is activated in the cells infected with WNV,^43^ expression of Rab27a may be increased as an antiviral inflammatory response.

Rab27a was not related to WNV genome replication despite suppressing viral multiplication. However, Rab27a was reported to promote replication of the HCV genome, a member of the *Flaviviridae* family.^26^ Rab27a has also been shown to support the assembly of HIV and human cytomegalovirus and the release of HCV, hepatitis E virus, and enterovirus A71 from cells.^21^^,^^22^^,^^23^^,^^24^^,^^25^ On the other hand, foot-and-mouth disease virus was shown to be negatively regulated by promotion of the exosome-mediated antiviral immune response by Rab27a.^44^ The findings of the present study and these previous studies suggested that Rab27a has various functions in cells infected with different viruses. The inhibitory effect of Rab27a on WNV multiplication observed in this study seemed to be the result of several factors.

This study showed that Rab27a inhibited WNV infection by mediating viral protein degradation in lysosomes. Investigation of the late stage of WNV infection, including viral transport, may be useful for the development of novel antiviral therapies. Rab proteins, including Rab27a, are involved in the transport of pathogens and tumor cell development, maturation, and migration.^45^^,^^46^^,^^47^^,^^48^^,^^49^ Further investigations and elucidation of functions of Rab proteins in virus infection may facilitate the development of novel therapies for various diseases.

### Limitations of the study

This study demonstrated the novel role of Rab27a in inhibiting WNV multiplication by mediating viral protein degradation in lysosomes. However, elucidation of the involvement of Rab27a in the pathogenesis of West Nile encephalitis in terms of drug development remains challenging. Further analyses using Rab27a knockout mice and drugs that regulate Rab27a expression will lead to a better understanding of the importance of Rab27a in the pathogenesis of encephalitis *in vivo*. Another limitation was the difficulty in identifying the pathway and original organelle of the vesicles containing viral proteins, due to the lack of appropriate equipment and materials. As experiments using live WNV must be performed at a BSL3 facility, further development of genetic manipulations of WNV is necessary to investigate the intracellular dynamics of WNV in detail.

## STAR★Methods

### Key resources table

**Table:** 

REAGENT or RESOURCE	SOURCE	IDENTIFIER
**Antibodies**		

Mouse anti-WNV E protein antibody	Murata et al.^55^	N/A
Rabbit anti-Rab27a polyclonal antibody	Proteintech	Cat# 17817-1-AP
Rabbit anti-LAMP1 polyclonal antibody	Abcam	Cat# ab24170
Mouse anti-WNV E protein monoclonal antibody	Millipore	Cat# MAB8151
HRP-conjugated b-actin monoclonal antibody	Proteintech	Cat# HRP-600008
Goat anti-Mouse IgG (H+L) Highly Cross-Adsorbed Secondary Antibody, Alexa Fluor Plus 488	Thermo Fisher Scientific	Cat# A32723
Goat anti-rabbit IgG (H+L) Highly Cross-Adsorbed Secondary Antibody, Alexa Fluor Plus 555	Thermo Fisher Scientific	Cat# A32732
Peroxidase AffiniPure Donkey Anti-Mouse IgG (H+L)	Jackson ImmunoResearch	Cat# 715-035-150
Peroxidase AffiniPure Donkey Anti-Rabbit IgG (H+L)	Jackson ImmunoResearch	Cat# 711-035-152

**Bacterial and virus strains**		

West Nile virus (NY99 6-LP strain)	Shirato et al.^50^	GenBank: AB185914

**Chemicals, peptides, and recombinant proteins**		

Bafilomycin A1	Wako	Cat# B0025
Cellstain Hoechst 33342	Wako	Cat# 041-30081
Lipofectamine 3000 Reagent	Thermo Fisher Scientific	Cat# L3000015
Lipofectamine RNAiMax Reagent	Thermo Fisher Scientific	Cat# 13778150
Lipofectamine MessengerMAX Reagent	Thermo Fisher Scientific	Cat# LMRNA008
PrimeScript One Step RT-PCR Kit Ver.2	Takara Bio	Cat# RR055A
Blasticidin S Hydrochloride	Wako	Cat# 029-18701
Protease inhibitor Cocktail	Nacalai Tesque	Cat# 25955-24

**Critical commercial assays**		

In-Fusion HD Cloning Kit	Takara Bio	Cat# 639649
Nano Glo luciferase assay substrate	Promega	Cat# N1120
mMESSAGE mMACHINE SP6 Transcription Kit	Thermo Fisher Scientific	Cat# AM1340
FlexAble CoraLite Plus 555 for Rabbit IgG	Proteintech	Cat# KFA002
FlexAble CoraLite Plus 405 for Rabbit IgG	Proteintech	Cat# KFA006

**Experimental models: Cell lines**		

Human: SH-SY5Y cells	European Collection of Authenticated cell cultures	# 94030304
African green monkey: Vero cells	JCRB Cell Bank	# JCRB0111
Human: 293T cells	Dr. Matsuura, Osaka University	N/A

**Oligonucleotides**		

siRNA for Rab27a #1:	Thermo Fisher Scientific	Cat# s11693
siRNA for Rab27a #2	Thermo Fisher Scientific	Cat# s11695

**Recombinant DNA**		

pCXSN-WNC	Kobayashi et al.^51^	N/A
pCMV-WNV-prME	Maezono et al.^52^	N/A
pCMV-WNrep-NLuc(sec)2A	This study	N/A
pWNrepNLuc(sec)2A	This study	N/A
CSII-CMV-MCS-IRES2-Bsd	Riken BioResource Center	# RDB04385
pCAG-HIVgp	Riken BioResource Center	# RDB04394
pCMV-VSV-G-Rev	Riken BioResource Center	# RDB04393
CSII-CMV-Rab27a-IRES2-Bsd	This study	N/A

**Software and algorithms**		

Image Lab Software	Bio-Rad	https://www.bio-rad.com/ja-jp/product/image-lab-software?ID=KRE6P5E8Z
Zen software	Zeiss	https://www.zeiss.com/microscopy/ja/products/software/zeiss-zen-lite.html
Fiji software	NIH	http://fiji.sc/Fiji

### Resource availability

#### Lead contact

Further information and requests for resources should be directed to and will be fulfilled by the lead contact, Shintaro Kobayashi (shin-kobayashi@vetmed.hokudai.ac.jp).

#### Materials availability

Plasmids generated in this study will be available upon request with a material transfer agreement (MTA).

#### Data and code availability

• All data reported in this paper will be shared by the lead contact upon request.
• This paper does not report original code.
• Any additional information required to reanalyze the data reported in this paper is available from the lead contact upon request.

### Experimental model and subject details

#### Cell lines

SH-SY5Y cells derived from human neuroblastoma were grown in Dulbecco’s modified Eagle medium (DMEM):Nutrient Mixture F-12 (Wako, Osaka, Japan) supplemented with 10% fetal bovine serum (FBS), 100 IU/mL penicillin, and 100 μg/mL streptomycin at 37°C with 5% CO_2_. Vero cells derived from African green monkey kidney epithelial cells were grown in Eagle’s minimum essential medium (MEM) (Wako), supplemented with 10% FBS, 100 IU/mL penicillin, and 100 μg/mL streptomycin at 37°C with 5% CO_2_. Human embryonic kidney 293T (293T) cells were cultured in high-glucose DMEM (Wako) supplemented with 10% FBS, 100 IU/mL penicillin, and 100 μg/mL streptomycin at 37°C with 5% CO_2_.

#### Virus

The WNV NY99 6-LP strain was established from the WNV NY99-6922 strain as described previously.^50^ Vero cells were infected with WNV and the supernatants were harvested at 3 days post-infection (dpi). The supernatants containing virus were centrifuged at 3000 × *g* for 5 min and stored at −80°C until use. All experiments using WNV were performed at the Biosafety Level 3 (BSL3) facility of Hokkaido University, Japan, in accordance with institutional guidelines.

### Methods details

#### Plasmid constructions and transfections

Plasmid encoding capsid protein (pCXSN-WNC) or prM and E proteins (pCMV-WNV-prME) were constructed previously.^51^^,^^52^ pCMV-WNrep-NLuc(sec)2A was constructed by introduction of the sequence of luciferase amplified by PCR from the plasmid pNL1.3 (Promega, Madison, WI, USA) into pCMV-WNrep-DsRed with removal of the DsRed sequence using an In-Fusion HD Cloning Kit (Takara Bio Inc., Shiga, Japan).^53^ pWNrepNLuc(sec)2A was generated from pWNrep-DsRed (generously provided by Dr. Akihiko Maeda, Kyoto Sangyo University, Kyoto, Japan) by replacing the DsRed sequence with the luciferase sequence.^54^ The lentiviral vector CSII-CMV-MCS-IRES2-Bsd and the packaging plasmids pCAG-HIVgp and pCMV-VSV-G-Rev were generously provided by Dr. Hiroyuki Miyoshi (Riken, Tsukuba, Japan). The sequence of Rab27a was amplified from total cellular RNA of SH-SY5Y cells using a PrimeScript One Step RT-PCR Kit Ver.2 (Takara Bio Inc.) and was introduced into CSII-CMV-MCS-IRES2-Bsd using an In-Fusion HD Cloning Kit (Takara Bio Inc.); the resultant construct was designated CSII-CMV-Rab27a-IRES2-Bsd.

The plasmids were transfected into cells by using Lipofectamine 3000 Transfection Reagent (Invitrogen, Waltham, MA, USA). Lipofectamine RNAiMax Transfection Reagent (Invitrogen) and Lipofectamine Messenger MAX Reagent (Invitrogen) were used for siRNA or RNA transfection, respectively.

#### Generation of SH-SY5Y cells stably expressing Rab27a

SH-SY5Y cells stably expressing Rab27a were established using lentiviral vectors. To construct lentiviral vectors, 293T cells were transfected with pCAG-HIVgp, pCMV-VSVG-RSV-Rev, and CSII-CMV-Rab27a-IRES2-Bsd or CSII-CMV-MCS-IRES2-Bsd. Supernatants were collected from the cells at 48 hpt and added to SH-SY5Y cells. The cells were incubated with growth medium containing 10 μg/mL blasticidin for selection of cells stably expressing Rab27a.

#### Immunoblotting

Cells were washed once with PBS and lysed in RIPA buffer consisting of 50 mM Tris (pH 8.0), 150 mM NaCl, 0.5% sodium deoxycholate, 0.1% SDS, and 1% Triton X-100 containing protease inhibitor (Nacalai Tesque, Kyoto, Japan) for 10 min on ice. The cell lysates were centrifuged at 17700 × *g* for 15 min at 4°C. The supernatants were mixed with an equal volume of 2× sample buffer consisting of 3% SDS, 10% glycerol, 100 mM Tris-HCl (pH 6.8), 0.1% bromophenol blue, and 10% 2-mercaptoethanol, and denatured at 95°C for 5 min. The cell lysates were separated by 12% SDS-PAGE and the proteins transferred onto PVDF membranes (Millipore). The membranes were blocked with 5% skim milk in TBS-T consisting of 0.1% Triton X-100, 25 mM Tris-HCl (pH 7.4), 132 mM NaCl, and 2.7 mM KCl, and incubated overnight at 4°C with the appropriate antibody. Bound antibodies were detected using HRP-conjugated secondary antibodies (Jackson ImmunoResearch, West Grove, PA, USA). The bands were visualized using the chemiluminescent HRP substrate (Millipore) and ChemiDoc XRS+ Imager (Bio-Rad, Hercules, USA), and the obtained images were analyzed using Image Lab Software (Bio-Rad).

#### Viral growth assay

SH-SY5Y cells transfected with siRNA for Rab27a or stably expressing Rab27a were infected with WNV at 1 or 0.5 plaque-forming units (pfu)/cell, respectively. The supernatants of WNV-infected cells were collected at 12 and 24 hpi and stored at −80°C until use. The diluted supernatants were inoculated into Vero cells. After 1-h incubation at 37°C with rocking, the inoculum was removed and the cells were incubated with overlay medium consisting of MEM containing 5% FBS and 1.25% methyl cellulose for 4 days. Plaques visualized using crystal violet solution (0.1% crystal violet, 70% ethanol, 10% formalin) were counted.

#### VLP release assay

For production of VLPs, SH-SY5Y cells into which siRNA had been introduced were transfected with pCXSN-WNC, pCMV-WNV-prME, and pCMV-WNrep-NLuc(sec)2A. The culture supernatants were collected at 48 hpt and inoculated into Vero cells. After 48 h, the cells were treated with Nano Glo luciferase assay substrate (Promega) for 3 min and luminescence was measured using GloMax (Promega).

#### Viral genome replication assay

pWNrep-Nluc(sec)2A was linearized with SalI and purified with phenol, isopropanol, and ethanol. The linearized plasmid was used to produce replicon RNA using an mMESSANGE mMACHINE SP6 Transcription Kit (Thermo Fisher Scientific) according to the manufacturer’s instructions. The replicon RNA was introduced into Rab27a-inhibited SH-SY5Y cells usign Lipofectamine Messenger MAX Reagent (Invitrogen). Luciferase activity was performed at 6, 12, or 24 hpt was measured using Nano Glo luciferase assay substrate (Promega).

#### Immunocytochemistry

The cells were fixed with 4% paraformaldehyde for 10 min at room temperature. Cells were permeabilized with PBST (PBS +0.1% Tween 20) for 10 min, blocked with PBS containing 1% BSA for 30 min, and stained with each antibody for overnight at 4°C. The cells were then washed with PBS and incubated with Alexa Fluor 488-conjugated anti-mouse IgG (Thermo Fisher Scientific) or Alexa Fluor 555-conjugated anti-rabbit IgG (Thermo Fisher Scientific) at room temperature for 1 h. The cells were observed by confocal microscopy (Zeiss LSM 800; Carl Zeiss, Jena, Germany), and the obtained images were analyzed using Zen software (Carl Zeiss) or Fiji software (http://fiji.sc/Fiji). More than three fields were observed in each experiment. The fluorescence intensity of Rab27a and E protein in each cell was measured. The pixel number of E protein or Rab27a colocalized with LAMP1 and that of whole E protein in each cell was measured and the colocalization ratio was calculated.

#### Lysosome inhibition assay

SH-SY5Y cells were infected with WNV at 1 pfu/cell and treated with 50 or 100 nM bafilomycin A1 for 3 h at 45 hpi. The cells were collected for immunocytochemistry or immunoblotting analysis. For immunocytochemistry, the cells were stained with mouse anti-WNV E protein antibody overnight at 4°C, followed by incubation with Alexa Fluor 488-conjugated anti-mouse IgG for 1 h at room temperature. The cells were stained with rabbit anti-Rab27a antibody labeled with FlexAble CoraLite Plus 555 antibody (Proteintech) and rabbit ani-LAMP1 antibody labeled with FlexAble CoraLite Plus 405 antibody (Proteintech) for overnight at 4°C.

### Quantification and statistical analysis

Data used in comparisons of two independent groups were evaluated in a normality test and compared in a either two-tailed Student’s *t* test or Mann−Whitney *U* test. Data for comparison among multiple groups were evaluated in a normality test and compared in a either the Tukey−Kramer test or Scheffe’s *F* test. All data are presented as the mean ± standard deviation. In all analyses, p < 0.05 was taken to indicate statistical significance.

## References

[1] Postler T.S., Beer M., Blitvich B.J., Bukh J., de Lamballerie X., Drexler J.F., Imrie A., Kapoor A., Karganova G.G., Lemey P. (2023). Renaming of the genus Flavivirus to Orthoflavivirus and extension of binomial species names within the family Flaviviridae. Arch. Virol..

[2] Chancey C., Grinev A., Volkova E., Rios M. (2015). The global ecology and epidemiology of West Nile virus. BioMed Res. Int..

[3] Kramer L.D., Ciota A.T., Kilpatrick A.M. (2019). Introduction, Spread, and Establishment of West Nile Virus in the Americas. J. Med. Entomol..

[4] Principi N., Esposito S. (2024). Development of Vaccines against Emerging Mosquito-Vectored Arbovirus Infections. Vaccines.

[5] Hackett B.A., Cherry S. (2018). Flavivirus internalization is regulated by a size-dependent endocytic pathway. Proc. Natl. Acad. Sci. USA.

[6] Vogt M.R., Dowd K.A., Engle M., Tesh R.B., Johnson S., Pierson T.C., Diamond M.S. (2011). Poorly neutralizing cross-reactive antibodies against the fusion loop of West Nile virus envelope protein protect in vivo via Fcgamma receptor and complement-dependent effector mechanisms. J. Virol..

[7] Chambers T.J., Hahn C.S., Galler R., Rice C.M. (1990). Flavivirus genome organization, expression, and replication. Annu. Rev. Microbiol..

[8] Fernandez-Garcia M.D., Mazzon M., Jacobs M., Amara A. (2009). Pathogenesis of flavivirus infections: using and abusing the host cell. Cell Host Microbe.

[9] Stenmark H. (2009). Rab GTPases as coordinators of vesicle traffic. Nat. Rev. Mol. Cell Biol..

[10] Banworth M.J., Li G. (2018). Consequences of Rab GTPase dysfunction in genetic or acquired human diseases. Small GTPases.

[11] Kiral F.R., Kohrs F.E., Jin E.J., Hiesinger P.R. (2018). Rab GTPases and Membrane Trafficking in Neurodegeneration. Curr. Biol..

[12] Krishnan M.N., Sukumaran B., Pal U., Agaisse H., Murray J.L., Hodge T.W., Fikrig E. (2007). Rab 5 is required for the cellular entry of dengue and West Nile viruses. J. Virol..

[13] Liu C.C., Zhang Y.N., Li Z.Y., Hou J.X., Zhou J., Kan L., Zhou B., Chen P.Y. (2017). Rab5 and Rab11 Are Required for Clathrin-Dependent Endocytosis of Japanese Encephalitis Virus in BHK-21 Cells. J. Virol..

[14] Fan J., Liu X., Mao F., Yue X., Lee I., Xu Y. (2020). Proximity proteomics identifies novel function of Rab14 in trafficking of Ebola virus matrix protein VP40. Biochem. Biophys. Res. Commun..

[15] Liu Y.Y., Bai J.S., Liu C.C., Zhou J.F., Chen J., Cheng Y., Zhou B. (2023). The Small GTPase Rab14 Regulates the Trafficking of Ceramide from Endoplasmic Reticulum to Golgi Apparatus and Facilitates Classical Swine Fever Virus Assembly. J. Virol..

[16] Kobayashi S., Suzuki T., Kawaguchi A., Phongphaew W., Yoshii K., Iwano T., Harada A., Kariwa H., Orba Y., Sawa H. (2016). Rab8b Regulates Transport of West Nile Virus Particles from Recycling Endosomes. J. Biol. Chem..

[17] Ostrowski M., Carmo N.B., Krumeich S., Fanget I., Raposo G., Savina A., Moita C.F., Schauer K., Hume A.N., Freitas R.P. (2010). Rab27a and Rab27b control different steps of the exosome secretion pathway. Nat. Cell Biol..

[18] Yi Z., Yokota H., Torii S., Aoki T., Hosaka M., Zhao S., Takata K., Takeuchi T., Izumi T. (2002). The Rab27a/granuphilin complex regulates the exocytosis of insulin-containing dense-core granules. Mol. Cell Biol..

[19] Yokoyama K., Kaji H., He J., Tanaka C., Hazama R., Kamigaki T., Ku Y., Tohyama K., Tohyama Y. (2011). Rab27a negatively regulates phagocytosis by prolongation of the actin-coating stage around phagosomes. J. Biol. Chem..

[20] Raposo G., Marks M.S., Cutler D.F. (2007). Lysosome-related organelles: driving post-Golgi compartments into specialisation. Curr. Opin. Cell Biol..

[21] Tamai K., Shiina M., Tanaka N., Nakano T., Yamamoto A., Kondo Y., Kakazu E., Inoue J., Fukushima K., Sano K. (2012). Regulation of hepatitis C virus secretion by the Hrs-dependent exosomal pathway. Virology.

[22] Nagashima S., Jirintai S., Takahashi M., Kobayashi T., Okamoto H., Tanggis, Nishizawa T., Kouki T., Yashiro T. (2014). Hepatitis E virus egress depends on the exosomal pathway, with secretory exosomes derived from multivesicular bodies. J. Gen. Virol..

[23] Wu J., Zhao Y., Chen Q., Chen Y., Gu J., Mao L. (2023). Enterovirus A71 Promotes Exosome Secretion by the Nonstructural Protein 3A Interacting with Rab27a. Microbiol. Spectr..

[24] Fraile-Ramos A., Cepeda V., Elstak E., van der Sluijs P. (2010). Rab27a is required for human cytomegalovirus assembly. PLoS One.

[25] Gerber P.P., Cabrini M., Jancic C., Paoletti L., Banchio C., von Bilderling C., Sigaut L., Pietrasanta L.I., Duette G., Freed E.O. (2015). Rab27a controls HIV-1 assembly by regulating plasma membrane levels of phosphatidylinositol 4,5-bisphosphate. J. Cell Biol..

[26] Chen T.C., Hsieh C.H., Sarnow P. (2015). Supporting Role for GTPase Rab27a in Hepatitis C Virus RNA Replication through a Novel miR-122-Mediated Effect. PLoS Pathog..

[27] Ramadass M., Catz S.D. (2016). Molecular mechanisms regulating secretory organelles and endosomes in neutrophils and their implications for inflammation. Immunol. Rev..

[28] Ménasché G., Pastural E., Feldmann J., Certain S., Ersoy F., Dupuis S., Wulffraat N., Bianchi D., Fischer A., Le Deist F., de Saint Basile G. (2000). Mutations in RAB27A cause Griscelli syndrome associated with haemophagocytic syndrome. Nat. Genet..

[29] Haddad E.K., Wu X., Hammer J.A., Henkart P.A. (2001). Defective granule exocytosis in Rab27a-deficient lymphocytes from Ashen mice. J. Cell Biol..

[30] Bobrie A., Krumeich S., Reyal F., Recchi C., Moita L.F., Seabra M.C., Ostrowski M., Théry C. (2012). Rab27a supports exosome-dependent and -independent mechanisms that modify the tumor microenvironment and can promote tumor progression. Cancer Res..

[31] Wang Q., Ni Q., Wang X., Zhu H., Wang Z., Huang J. (2015). High expression of RAB27A and TP53 in pancreatic cancer predicts poor survival. Med. Oncol..

[32] Blanc L., Vidal M. (2018). New insights into the function of Rab GTPases in the context of exosomal secretion. Small GTPases.

[33] Shimada-Sugawara M., Sakai E., Okamoto K., Fukuda M., Izumi T., Yoshida N., Tsukuba T. (2015). Rab27A regulates transport of cell surface receptors modulating multinucleation and lysosome-related organelles in osteoclasts. Sci. Rep..

[34] Laulagnier K., Schieber N.L., Maritzen T., Haucke V., Parton R.G., Gruenberg J. (2011). Role of AP1 and Gadkin in the traffic of secretory endo-lysosomes. Mol. Biol. Cell.

[35] Fukuda M. (2005). Versatile role of Rab27 in membrane trafficking: focus on the Rab27 effector families. J. Biochem..

[36] Bhuin T., Roy J.K. (2014). Rab proteins: the key regulators of intracellular vesicle transport. Exp. Cell Res..

[37] Xu X.F., Chen Z.T., Zhang J.L., Chen W., Wang J.L., Tian Y.P., Gao N., An J. (2008). Rab8, a vesicular traffic regulator, is involved in dengue virus infection in HepG2 cells. Intervirology.

[38] Allison S.L., Stadler K., Mandl C.W., Kunz C., Heinz F.X. (1995). Synthesis and secretion of recombinant tick-borne encephalitis virus protein E in soluble and particulate form. J. Virol..

[39] Sasaki M., Anindita P.D., Phongphaew W., Carr M., Kobayashi S., Orba Y., Sawa H. (2018). Development of a rapid and quantitative method for the analysis of viral entry and release using a NanoLuc luciferase complementation assay. Virus Res..

[40] Dornier E., Rabas N., Mitchell L., Novo D., Dhayade S., Marco S., Mackay G., Sumpton D., Pallares M., Nixon C. (2017). Glutaminolysis drives membrane trafficking to promote invasiveness of breast cancer cells. Nat. Commun..

[41] Zhong W., Lu Y., Han X., Yang J., Qin Z., Zhang W., Yu Z., Wu B., Liu S., Xu W. (2023). Upregulation of exosome secretion from tumor-associated macrophages plays a key role in the suppression of anti-tumor immunity. Cell Rep..

[42] Feng F., Jiang Y., Lu H., Lu X., Wang S., Wang L., Wei M., Lu W., Du Z., Ye Z. (2016). Rab27A mediated by NF-kappaB promotes the stemness of colon cancer cells via up-regulation of cytokine secretion. Oncotarget.

[43] Scherbik S.V., Pulit-Penaloza J.A., Basu M., Courtney S.C., Brinton M.A. (2013). Increased early RNA replication by chimeric West Nile virus W956IC leads to IPS-1-mediated activation of NF-kappaB and insufficient virus-mediated counteraction of the resulting canonical type I interferon signaling. J. Virol..

[44] Xu G., Xu S., Shi X., Shen C., Zhang D., Zhang T., Hou J., Zhang K., Zheng H., Liu X. (2020). Foot-and-mouth disease virus degrades Rab27a to suppress the exosome-mediated antiviral immune response. Vet. Microbiol..

[45] Marsh M., Helenius A. (2006). Virus entry: open sesame. Cell.

[46] Smith A.C., Heo W.D., Braun V., Jiang X., Macrae C., Casanova J.E., Scidmore M.A., Grinstein S., Meyer T., Brumell J.H. (2007). A network of Rab GTPases controls phagosome maturation and is modulated by Salmonella enterica serovar Typhimurium. J. Cell Biol..

[47] Bravo-Cordero J.J., Marrero-Diaz R., Megías D., Genís L., García-Grande A., García M.A., Arroyo A.G., Montoya M.C. (2007). MT1-MMP proinvasive activity is regulated by a novel Rab8-dependent exocytic pathway. EMBO J..

[48] Cheng K.W., Lahad J.P., Kuo W.L., Lapuk A., Yamada K., Auersperg N., Liu J., Smith-McCune K., Lu K.H., Fishman D. (2004). The RAB25 small GTPase determines aggressiveness of ovarian and breast cancers. Nat. Med..

[49] Hou Q., Wu Y.H., Grabsch H., Zhu Y., Leong S.H., Ganesan K., Cross D., Tan L.K., Tao J., Gopalakrishnan V. (2008). Integrative genomics identifies RAB23 as an invasion mediator gene in diffuse-type gastric cancer. Cancer Res..

[50] Shirato K., Miyoshi H., Goto A., Ako Y., Ueki T., Kariwa H., Takashima I. (2004). Viral envelope protein glycosylation is a molecular determinant of the neuroinvasiveness of the New York strain of West Nile virus. J. Gen. Virol..

[51] Kobayashi S., Yoshii K., Phongphaew W., Muto M., Hirano M., Orba Y., Sawa H., Kariwa H. (2020). West Nile virus capsid protein inhibits autophagy by AMP-activated protein kinase degradation in neurological disease development. PLoS Pathog..

[52] Maezono K., Kobayashi S., Tabata K., Yoshii K., Kariwa H. (2021). Development of a highly specific serodiagnostic ELISA for West Nile virus infection using subviral particles. Sci. Rep..

[53] Kobayashi S., Yoshii K., Hirano M., Muto M., Kariwa H. (2017). A novel reverse genetics system for production of infectious West Nile virus using homologous recombination in mammalian cells. J. Virol. Methods.

[54] Maeda J., Takagi H., Hashimoto S., Kurane I., Maeda A. (2008). A PCR-based protocol for generating West Nile virus replicons. J. Virol. Methods.

[55] Murata R., Eshita Y., Maeda A., Maeda J., Akita S., Tanaka T., Yoshii K., Kariwa H., Umemura T., Takashima I. (2010). Glycosylation of the West Nile Virus envelope protein increases in vivo and in vitro viral multiplication in birds. Am. J. Trop. Med. Hyg..

